# Exploiting the biogenesis of extracellular vesicles for bioengineering and therapeutic cargo loading

**DOI:** 10.1016/j.ymthe.2023.02.013

**Published:** 2023-02-20

**Authors:** Julia Rädler, Dhanu Gupta, Antje Zickler, Samir EL Andaloussi

**Affiliations:** 1 Biomolecular Medicine, Division of Biomolecular and Cellular Medicine, Department of Laboratory Medicine, Karolinska Institutet, 141 57 Huddinge, Sweden; 2 Department of Paediatrics, University of Oxford, Oxford OX3 9DU, UK

**Keywords:** exosomes, microvesicles, drug delivery, EV heterogeneity, EV engineering

## Abstract

Extracellular vesicles (EVs) are gaining increasing attention for diagnostic and therapeutic applications in various diseases. These natural nanoparticles benefit from favorable safety profiles and unique biodistribution capabilities, rendering them attractive drug-delivery modalities over synthetic analogs. However, the widespread use of EVs is limited by technological shortcomings and biological knowledge gaps that fail to unravel their heterogeneity. An in-depth understanding of their biogenesis is crucial to unlocking their full therapeutic potential. Here, we explore how knowledge about EV biogenesis can be exploited for EV bioengineering to load therapeutic protein or nucleic acid cargos into or onto EVs. We summarize more than 75 articles and discuss their findings on the formation and composition of exosomes and microvesicles, revealing multiple pathways that may be stimulation and/or cargo dependent. Our analysis further identifies key regulators of natural EV cargo loading and we discuss how this knowledge is integrated to develop engineered EV biotherapeutics.

## Graphical abstract

**Figure:**
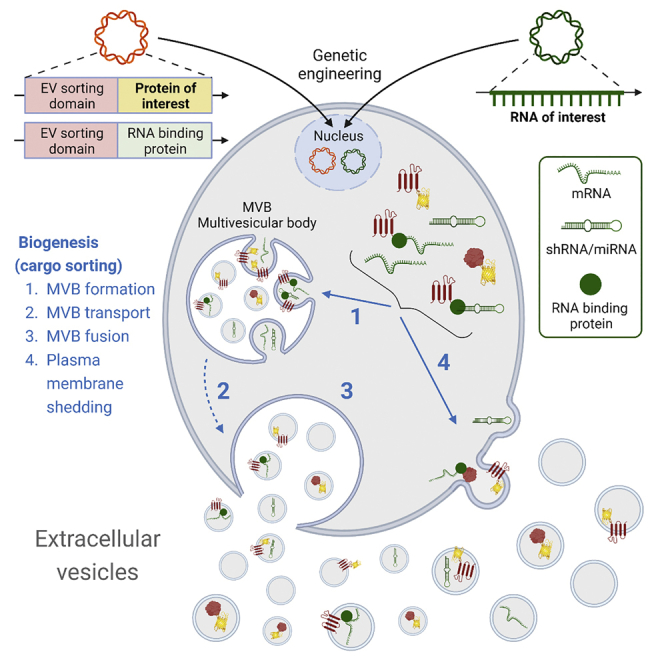

## Teaser

A lack of biological knowledge and technical challenges limits the widespread therapeutic application of extracellular vesicles. This review summarizes current knowledge on the biogenesis and composition of exosomes and microvesicles. Moreover, it extends into natural cargo sorting and discusses how this knowledge can be harnessed to develop engineered EV biotherapeutics.

## Introduction

Extracellular vesicles (EVs) are natural membrane-enclosed nanoparticles secreted by all cells. They are important mediators of intercellular communication that convey messages to surrounding or distant cells in the form of proteins, lipids, nucleic acids,^1^^,^^2^ and even organelles.^3^^,^^4^^,^^5^^,^^6^ Thus, EVs exert not only physiological but also pathological functions, which renders them attractive therapeutic targets.^7^^,^^8^ Correspondingly, they serve as diagnostic biomarkers, mainly for cancer and neurodegenerative diseases.^8^ Last, owing to their unique biodistribution capabilities, versatility, and immune tolerance, EVs are being increasingly exploited as drug-delivery vehicles.^9^ However, their widespread application is partially limited by technical challenges in addressing EV heterogeneity.^8^ These issues encompass things from the choice of producer cell to the methods of EV isolation and characterization.^9^^,^^10^ Another similar concern sometimes is a lack of robust cargo loading strategies that rely on the biogenesis or composition of EVs.

Classically, EVs are divided into three types of vesicles based on their origin: (1) exosomes (50–200 nm), formed in the endolysosomal system^11^ or at the plasma membrane^12^; (2) microvesicles or ectosomes (0.1–1 μm), budding directly from the plasma membrane^13^^,^^14^^,^^15^; and (3) apoptotic bodies (1–5 μm) generated by dying cells.^16^ Since the last are recognized and removed by macrophages,^17^ it is mainly exosomes and microvesicles that are of therapeutic interest and thus will be the focus of this review. To date, the field has struggled to physically separate these two classes of EVs because they overlap in size and composition. They even share commonalities in their biogenesis pathways, which complicates the definition of mutually exclusive properties for characterization.^18^ However, owing to technological advancements, increasingly more is known about the mechanisms that govern their biogenesis and determine their composition. In addition, understanding the underlying processes is crucial for harnessing them as biotherapeutics.

## Biogenesis of EVs

**Figure 1:**
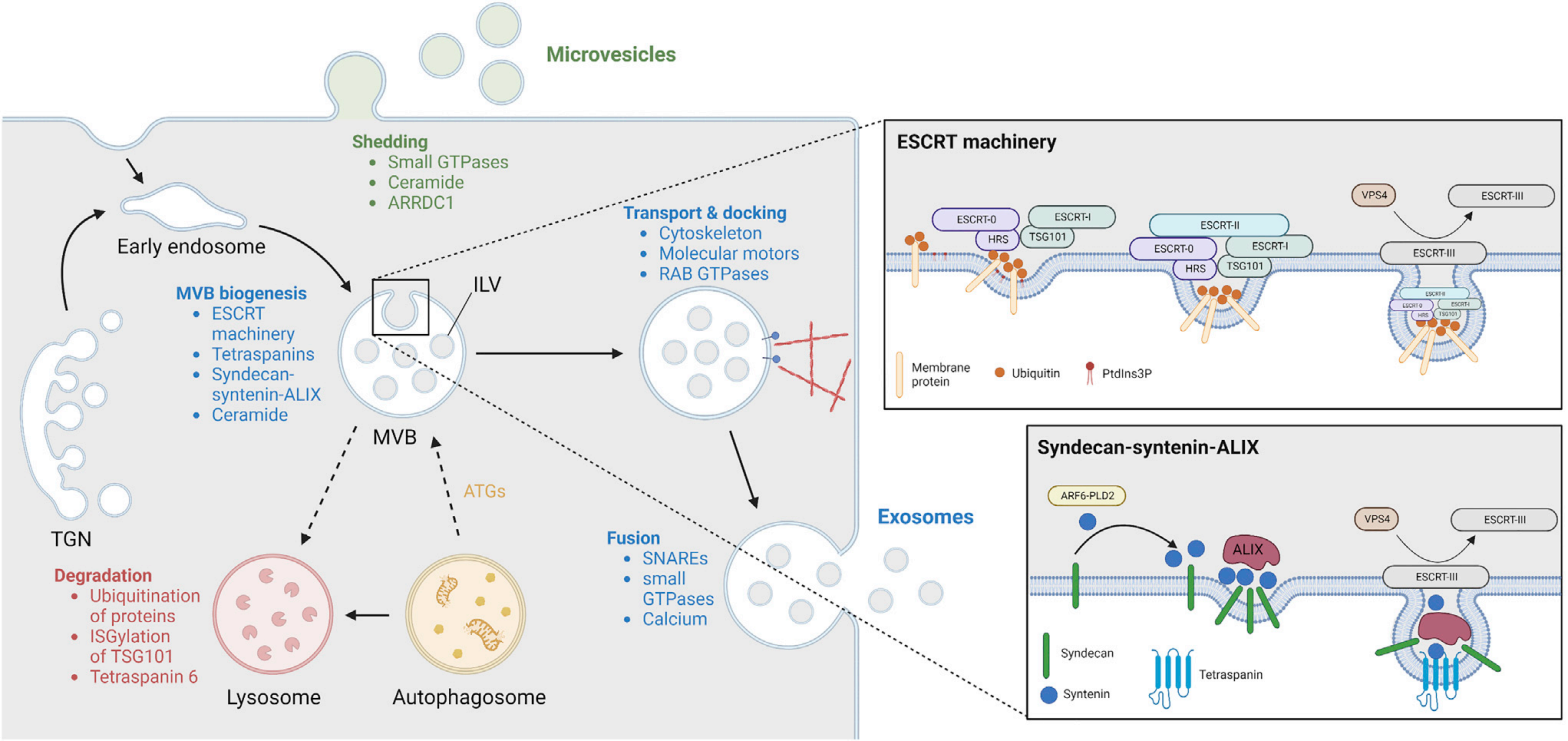
EV biogenesis and its regulators The shedding of microvesicles from the plasma membrane is controlled by small GTPases, ceramide, or ARRDC1. Exosome biogenesis is a more complex process taking place directly at the plasma membrane or in the endolysosomal system. Cargo is sorted into endosomes from the plasma membrane or the TGN. The early endosome then matures and starts forming ILVs, thereby becoming an MVB. ILV formation can be regulated by the ESCRT machinery, tetraspanins, syndecan-syntenin-ALIX, and ceramide. (Top right) ESCRT machinery: HRS (subunit of ESCRT-0) recognizes ubiquitinated proteins and PtdIns3P, leading to the recruitment of ESCRT-I by binding of its TSG101 subunit to HRS. ESCRT-II then drives the invagination and ESCRT-III the scission of the membrane. Reuse of ESCRT-III is enabled by VPS4. (Bottom right) Syndecan-syntenin-ALIX: syndecan recruits syntenin, which is regulated by ARF6 and PLD2. Subsequent interaction with ALIX promotes ILV formation, which is finalized by ESCRT-III and VPS4. This pathway commonly involves tetraspanins. After their formation, MVBs can fuse with lysosomes for degradation, which is promoted by ubiquitination of proteins, ISGylation of TSG101, and tetraspanin 6. ATGs, on the other hand, can facilitate MVB biogenesis. For secretion, MVBs are transported to the plasma membrane with the help of cytoskeletal elements, molecular motors, and RABs. Ultimately, fusion with the plasma membrane leading to the release of exosomes is mediated by SNAREs, small GTPases, and calcium. This figure was created using BioRender. ARF6, ADP ribosylation factor 6; ARRDC1, arrestin-domain-containing protein 1; ATG, autophagy-related protein; ESCRT, endosomal sorting complexes required for transport; HRS, hepatocyte growth factor-regulated tyrosine kinase substrate; ILV, intraluminal vesicle; ISG, interferon-stimulated gene; MVB, multivesicular body; PLD2, phospholipase D2; PtdIns3P, phosphatidylinositol 3-phosphate; RAB, Ras-associated binding protein; SNARE, soluble N-ethylmaleimide-sensitive factor attachment protein receptor; TGN, *trans*-Golgi network; TSG101, tumor susceptibility gene 101; VPS4, vacuolar protein sorting-associated protein 4.

The biogenesis of EVs relies on intricate processes that are mediated by a complex interplay of signaling molecules and regulators (Figure 1). In Table 1 we compare 77 studies focusing on the regulators of different steps in EV biogenesis. For each study, we list the EV regulator of interest along with the examined producer cell types and, if relevant, applied stimuli. Moreover, we state the method of intervention with the EV regulator, the investigated EV population, and, ultimately, the impact the intervention had on EV biogenesis as measured predominantly by EV production (Table 1). The implications of the findings of these studies are discussed in more detail below. The overarching goal of this analysis was to provide a comprehensive overview of known regulators of EV biogenesis and, eventually, their putative role in EV bioengineering as discussed in the later sections of this review.

**Table 1:** Regulators of EV biogenesis

Regulator	Cell	Intervention	EVs studied	Effect	Reference
Exosomes					

Endosome maturation					
RAB5	MCF7	overexpression (RAB5^Q79L^)	Bulk	↓ EV secretion (syndecan, CD63, syntenin, ALIX)	Baietti et al.^19^
	mOli-neu + PLP	overexpression (RAB5^Q79L^)	Bulk	↓ EV release of PLP	Trajkovic et al.^20^
RAB7	MCF7	RNAi	Bulk	↓ EV secretion (syndecan, CD63, syntenin)	Baietti et al.^19^

MVB formation					

ESCRT machinery					
ESCRT-0					
- HRS	HeLa-CIITA	RNAi	CD63 (CD81^+^/MHCII^+^)	↓ EV production	Colombo et al.^21^
	stimulated mDCs	RNAi	Bulk	↓ EV production	Tamai et al.^22^
	mBMDCs	RNAi	Bulk	↓ EV production	Tamai et al.^22^
	SCC25-H1047R	RNAi	Bulk	↓ EV production	Hoshino et al.^23^
	HeLa	RNAi	N/A	↑ MVB diameter	Edgar et al.^24^
				↓ ILVs per MVB	
	A431	RNAi	N/A	↓ ILVs per MVB area	Razi and Futter^25^
- STAM1	HeLa-CIITA	RNAi	CD63 (CD81^+^/MHCII^+^)	↓ EV production	Colombo et al.^21^
ESCRT-I					
- TSG101	HeLa-CIITA	RNAi	CD63 (CD81^+^/MHCII^+^)	↓ EV production	Colombo et al.^21^
	HeLa	RNAi	N/A	↑ MVB diameter	Edgar et al.^24^
				↓ ILVs per MVB	
	A431	RNAi	Na	↓ MVB formation	Razi and Futter^25^
ESCRT-III					
- CHMP6	HeLa GFP-CHMP4B	RNAi	bulk	↓ EV proteins (CD9, CD63, CD81, syntenin)	Larios et al.^26^
VPS4B	HeLa-CIITA	RNAi	CD63 (CD81^+^/MHCII^+^)	↑ EV production	Colombo et al.^21^
	HeLa	overexpression (hVPS4^E223Q^)	N/A	↓ ILVs per MVB	Sachse et al.^27^
Tetraspanins					
CD82/CD9	HEK293T	overexpression (CD82/CD9)	bulk	↑ EV production; EV β-catenin	Chairoungdua et al.^28^
CD9	HEK293 CD9^−/−/-^	overexpression (CD9^YEVM^)^a^	bulk	↓ EV protein (CD9)	Fordjour et al.^12^
CD63	MNT-1	RNAi	N/A	↓ ILVs per MVB	van Niel et al.^29^
	HEK293	KO	bulk	↓ particles per cell	Hurwitz et al.^30^
	HEK293 CD63^−/−^	overexpression (CD63^Y235A^)	bulk	↑ EV protein (CD63)	Fordjour et al.^12^
Syndecan-syntenin-ALIX					
Syndecan	MCF7	RNAi	bulk	↓ EV production	Baietti et al.^19^
Syntenin	MCF7	RNAi	bulk	↓ EV production	Baietti et al.^19^
	MCF7	Overexpression	bulk	↑ EV production	Baietti et al.^19^
- ARF6	MCF7	RNAi	bulk	↓ EV proteins (syntenin, ALIX, CD63, SDC1CTF)	Ghossoub et al.^31^
	MCF7	overexpression (ARF6^T157N^)	bulk	↑ EV proteins (syntenin, ALIX, CD63)	Ghossoub et al.^31^
-- PLD2	MCF7	RNAi	bulk	↓ EV proteins (syntenin, ALIX, CD63)	Ghossoub et al.^31^
ALIX	HeLa-CIITA	RNAi	CD63 (CD81^+^/MHCII^+^)	↑ MHCII on EVs	Colombo et al.^21^
	MCF7	RNAi	bulk	↓ EV production	Baietti et al.^19^
	HeLa GFP-CHMP4B	overexpression (ALIXΔPRR)	bulk	↑ EV proteins (CD9, CD63, CD81)	Larios et al.^26^
	HeLa GFP-CHMP4B	RNAi	bulk	↓ EV proteins (CD9, CD63, CD81, syntenin)	Larios et al.^26^
Ceramide					
nSMase2	mOli-neu + PLP	GW4869/spiroepoxide/glutathione/RNAi	bulk	↓ EV release of PLP	Trajkovic et al.^20^
	HEK293T + CD82	GW4869	bulk	↓ EV proteins (flotillin, β-catenin)	Chairoungdua et al.^28^
	SCC25-H1047R	GW4869	bulk	↓ EV production	Hoshino et al.^23^
	HEK293T	GW4869/RNAi	bulk	↓ EV production	Leidal et al.^32^
				↓ EV proteins (LC3, SAFB, HNRNPK)	
	HeLa FLAG-RAB31^Q65L^	GW4869	bulk	↓ EV proteins (FLAG, EGFR, FLOT1, FLOT2, CD9, CD81, CD63)	Wei et al.^33^
ATGs					
ATG5	MEF, MDA-MB-231	KO	bulk	↓ EV production	Guo et al.^34^
				↓ EV proteins (Flotillin2, Tsg101)	
ATG12-ATG3	MEF	overexpression (ATG3^K243R^)	bulk	↓ EV proteins (total; ALIX, TSG101, GAPDH)	Murrow et al.^35^

Transport					

Cortactin	SCC61	RNAi	bulk	↓ EV production	Sinha et al.^36^
				↓ EV proteins (TSG101, CD63, Flotillin1)	
	SCC61	Overexpression	bulk	↑ EV production	Sinha et al.^36^
RAB GTPases					
RAB11	K562	overexpression (RAB11^S25N^)	bulk	↓ EV proteins (TfR, Lyn, Hsc70)	Savina et al.^37^
				↓ EV AChE activity	
RAB35	mOli-neu + PLP	overexpression (RAB35^N120I^), RNAi	bulk	↓ EV release of PLP	Hsu et al.^38^
RAB27A, RAB27B	HeLa	RNAi	bulk	↓ EV production	Ostrowski et al.^39^
				↓ EV proteins (HLA-DR, CD63, TSG101, HSC70)	
RAB27A	B16-F10,SK-Mel-28	RNAi	bulk	↓ EV protein (total)	Peinado et al.^40^
RAB27A	TS/A, 4T1	RNAi	bulk	↓ EV proteins (total; ALIX, HSC70, CD63, TSG101)	Bobrie et al.^41^
Fusion					
VAMP7	K562	overexpression (NT-VAMP7)	bulk	↓ EV production	Fader et al.^42^
				↓ EV AChE activity	
YKT6	HEK293	RNAi	bulk	↓ EV proteins (WNT3A, CD81)	Gross et al.^43^
	A549	RNAi	bulk	↓ EV protein (TSG101)	Ruiz-Martinez et al.^44^
SNAP23	A549	RNAi	bulk	↓ EV production	Wei et al.^45^
	HeLa + histamine + CD63-pHluorin	RNAi	CD63-pHluorin	↓ fusion activity	Verweij et al.^46^
Syntaxin-4	HeLa + histamine + CD63-pHluorin	RNAi	CD63-pHluorin	↓ fusion activity	Verweij et al.^46^
RAL1	4T1	RNAi	bulk	↓ EV production	Hyenne et al.^47^
				↓ EV proteins (ALIX, CD63, TSG101, HSC70)	
RalA/B	4T1	RNAi	bulk	↓ EV production	Ghoroghi et al.^48^
Calcium	K562	monensin	bulk	↑ EV protein (TfR, Hsc70)	Savina et al.^49^^,^^50^

↑ EV AChE activity

Microvesicles					

ARF6	LOX^ARF6-GTP^	overexpression (ARF6^Q67L^)	bulk	↑ EV protein (total)	Muralidharan-Chari et al.^51^
RhoA	HeLa + EGF	overexpression (RhoA^F30L^)	bulk	↑ EV production	Li et al.^52^
				↑ GFP release in EVs	
ARF1	MDA-MB-231	RNAi	bulk	↓ EV protein (total)	Schlienger et al.^53^
				↓ EV MMP9 activity	
ASM	astrocytes	imipramine	bulk	↓ EV fluorescence	Bianco et al.^54^
	N9	r-SMase	bulk	↑ EV fluorescence	Bianco et al.^54^
	RBCs	amitriptyline	Annexin V	↓ EV percentage	Awojoodu et al.^55^
ARRDC1	HEK293T	overexpression (ARRDC1-GFP)	bulk	↑ GFP release in EVs	Nabhan et al.^56^

For each study, the producer cell type and, if relevant, any applied stimuli are mentioned. Moreover, the method of intervention, the investigated EV population, and the impact the intervention had on EV biogenesis are stated. EVs were predominantly studied in bulk and only a few studies looked at specific subpopulations.

Regulators: ALIX, programmed cell death 6-interacting protein; ARF, ADP ribosylation factor; ARRDC1, arrestin-domain-containing protein 1; ASM, acid sphingomyelinase; ATG, autophagy-related protein; CHMP, charged multivesicular body protein; ESCRT, endosomal sorting complexes required for transport; HRS, hepatocyte growth factor-regulated tyrosine kinase substrate; nSMase2, neutral sphingomyelinase 2; PLD, phospholipase D; RAB, Ras-associated binding protein; RAL, Ras-like protein; Rho, Ras homologous protein; SNAP, synaptosomal-associated protein; STAM1, signal-transducing adaptor molecule 1; TSG101, tumor susceptibility gene 101; VAMP, vesicle-associated membrane protein; VPS4B, vacuolar protein sorting-associated protein 4B; YKT6, synaptobrevin homolog. Cells: MCF-7, human breast cancer cell line; 4T1, murine breast cancer cell line; A431, human epidermoid squamous carcinoma cell line; B16-F10, murine melanoma cell line; CIITA, major histocompatibility complex class II transactivator; HEK, human embryonic kidney cell line; HeLa, human cervical cancer cell line; K562, human myelogenous leukemia cell line; LOX, human melanoma cell line; mBMDCs, murine bone marrow-derived dendritic cells; MDA-MB-231, human breast cancer cell line; mDCs, murine dendritic cells; MEF, mouse embryonic fibroblasts; mOli-neu, murine oligodendroglial precursor cell line; N9, microglial cell line; RBCs, red blood cells; SCC, squamous cell carcinoma cell line; SK-MEL-28, human melanoma cell line; TS/A, murine mammary adenocarcinoma cell line. Others: ↓, reduction; ↑, increase; AChE, acetylcholinesterase; EGFR, epidermal growth factor receptor; FLOT, flotillin; GAPDH, glyceraldehyde-3-phosphate dehydrogenase; HLA-DR, human leukocyte antigen DR; HNRNPK, heterogeneous nuclear ribonucleoprotein K; HSC70, heat-shock 70 kDa protein; ILV, intraluminal vesicle; KO, knockout; LC3, microtubule-associated protein 1A/1B-light chain 3; Lyn, tyrosine-protein kinase; MHCII, major histocompatibility class II; MMP9, metalloproteinase 9; MV, microvesicle; MVB, multivesicular body; N/A, not applicable; PLP, proteolipid protein; RNAi, RNA interference; r-SMase, recombinant sphingomyelinase; SAFB, scaffold-attachment factor B; SDC1CTF, syndecan-1 cytoplasmic fragment; TfR, transferrin receptor; WNT3A, Wnt family member 3A.

a CD9 mutant that carries endosome-targeting signal.

## Exosomes

Only recently, Pegtel and Gould challenged the common notion that exosome-sized vesicles stem primarily from the endolysosomal system.^11^ Indeed, Fordjour et al. showed experimentally that efficient budding from exosomes can occur at the plasma membrane,^12^ which was recently confirmed independently.^57^ Given the novelty of these observations, pre-existing literature considered typically only endolysosomal exosome biogenesis, which begins with the formation of multivesicular bodies (MVBs), which are then transported to the plasma membrane and, upon fusion, release exosomes into the extracellular space (Figure 1).^1^ MVBs are formed during endosome maturation, which is mediated by Ras-associated binding (RAB) GTPases, particularly the conversion of RAB5 to RAB7 (Table 1).^58^^,^^59^^,^^60^ During the transition from early to late endosomes, the limiting membrane invaginates to give rise to intraluminal vesicles (ILVs). Multiple drivers of this process have been identified, including the endosomal sorting complex required for transport (ESCRT) machinery (Table 1).^61^ This machinery consists of four subunits (ESCRT-0, -I, -II, and -III) that act in succession. HRS, a component of ESCRT-0, is the first to engage with the endosomal membrane, where it interacts with ubiquitinated proteins^62^^,^^63^ and binds to phosphoinositide (PtdIns3P).^64^^,^^65^^,^^66^ Next, ESCRT-I is recruited by binding of its TSG101 subunit to HRS.^67^^,^^68^ ESCRT-II assembly follows, and ESCRT-II, together with ESCRT-I, drives the invagination of the endosomal membrane.^69^^,^^70^^,^^71^ Recruitment of ESCRT-III results in membrane scission, thereby finalizing ILV formation.^72^^,^^73^ To enable reuse, disassociation of ESCRT-III requires VPS4 (Figure 1).^74^^,^^75^^,^^76^ Importantly, depletion of the ESCRT machinery does not abolish exosome production, demonstrating that MVBs can also be formed by other means.^77^

Alternative processes for MVB formation have been proposed; however, their cross talk and interdependence are still understudied. Tetraspanins, particularly CD63, CD81, and CD9, have emerged as key regulators of alternative MVB formation processes (Table 1). They are involved in ILV formation by clustering together and sequestering other proteins to form tetraspanin-enriched microdomains.^78^^,^^79^ CD63, for instance, was found to compete with HRS in ILV formation,^24^ implying that the production of tetraspanin-containing exosomes does not require the canonical ESCRT machinery.^29^^,^^33^ In fact, CD63 has been shown to regulate exosome production,^29^^,^^30^ and the cone-like shape of CD9 has been proposed to aid membrane curvature.^80^ However, increasing evidence suggests that tetraspanin-dependent exosome biogenesis requires other regulators, such as ALIX, ESCRT-III,^26^ or syntenin.^33^ This so-called syndecan-syntenin-ALIX pathway utilizes ESCRT-III and VPS4 but does not require ESCRT-0 or ubiquitination.^81^ Instead, syndecan recruits syntenin, which then interacts with ALIX to promote ILV biogenesis.^19^^,^^82^ Syntenin, in turn, was shown to be regulated by ARF6 and phospholipase D2 (PLD2) (Figure 1).^31^ In another study, however, exosomal secretion of β-catenin driven by CD82 or CD9 overexpression was dependent on ceramide,^28^ which is another known regulator of ILV formation. Briefly, neutral sphingomyelinase (nSMase) 2 hydrolyzes sphingomyelin to ceramide, which initiates membrane curvature. Multiple studies have shown that inhibition of sphingomyelinase reduces exosome release,^20^^,^^23^^,^^28^^,^^32^^,^^83^^,^^84^^,^^85^ while induction with ceramide causes an increase (Table 1).^20^^,^^85^^,^^86^ Recently, non-conventional exosome production at the nuclear envelope of activated neutrophils was found to require nSMase1 and ceramide.^87^ In addition, interaction of nSMase activation-associated factor (NSMAF), a regulator of nSMase2, with microtubule-associated protein 1A/1B-light chain 3 (LC3) is pivotal for its loading and secretion in ceramide-dependent exosomes.^32^ Also, ceramide-dependent formation of EGFR-containing exosomes requires RAB31 to engage flotillin proteins.^33^ Intriguingly, Wei et al. hypothesize that RAB31-flotillin regulates tetraspanin sorting into exosomes, which upon stimulation (e.g., EGFR) predominates over the basal syndecan-syntenin-ALIX pathway.^33^ Clearly, MVB formation is a highly complex process that appears cargo and stimulation dependent and involves redundant pathways.

After their formation, MVBs either are destined for degradation in lysosomes or fuse with the plasma membrane, leading to the release of ILVs as exosomes (Figure 1). The drivers of this decision are largely unknown, but are believed to be determined already during MVB formation. For instance, ubiquitination of proteins,^88^^,^^89^ ISGylation of TSG101,^90^ or tetraspanin-6, a negative regulator of syntenin,^91^ have been shown to lead MVBs down the degradative pathway. This supports the notion that ESCRT-dependent formation of MVBs is commonly associated with degenerative and syndecan-syntenin-ALIX with secretory MVBs.^19^^,^^92^ Also, lysobisphosphatidic acid (LBPA), a partner of ALIX, has been proposed to regulate the fate of ILVs.^26^^,^^81^^,^^93^^,^^94^ Aside from that, unconventional protein secretion from autophagosomes can involve MVBs.^95^ In fact, autophagy-related (ATG) proteins have been implicated in exosome production (Table 1) by decreasing the acidification of MVBs^34^ or by interacting with and stimulating ALIX.^35^

The transport of MVBs to the plasma membrane is largely unknown but shares mechanisms of conventional vesicular trafficking along cytoskeletal elements. Hence, it is an active process driven by molecular motors and directed by small GTPases (i.e., RAB GTPases). The involvement of cytoskeletal proteins was exemplified in a study where exosome-mediated transfer of CD63-GFP from polarized T cells to antigen-presenting cells (APCs) was abolished in the presence of actin cytoskeleton inhibitors.^96^ Furthermore, cortactin, as a regulator of branched actin dynamics, was found to control MVB docking at the plasma membrane.^36^ Also, ALIX has been suggested to play a role in actin-dependent intracellular distribution of endosomes.^97^ Apart from that, RAB GTPases have been identified as spatiotemporal coordinators of MVB traffic (Table 1). Initially, RAB11 was found to regulate exosome release in K562 cells^37^ and has since been shown to do so by influencing plasma membrane docking of MVBs,^49^ similar to RAB35 in oligodendroglial cells.^38^ In addition, RAB27 isoforms have been observed to function differently depending on the cell type (Table 1). Similarly, in contrast to K562 cells,^37^ RAB11 inhibition did not affect the production of exosomes in HeLa cells,^39^ supporting the overall notion that RAB GTPases regulate exosome secretion in a cell-type-dependent manner.

The fusion of MVBs with the plasma membrane and release of exosomes are driven by soluble N-ethylmaleimide-sensitive factor attachment protein receptor (SNARE) proteins. Here, vesicle-associated membrane proteins (VAMPs; or v-SNAREs) interact with syntaxin and SNAP (or t-SNAREs) on the plasma membrane to form *trans*-SNARE complexes, which provide proximity and the necessary mechanical force.^98^ SNAREs involved in exosome release include VAMP7,^99^^,^^42^ YTK6,^43^^,^^44^ SNAP23,^45^^,^^46^ and syntaxin-4 (Table 1).^46^ Their downregulation negatively affects exosome release rates, but in the case of VAMP7, only in K562^42^ and not in MDCK cells.^99^ Other proteins involved in exosome secretion include V-ATPases^100^ and small GTPases. Intriguingly, Verweij et al. developed a pH-sensitive system using CD63-pHluorin to directly visualize fusion of MVBs with the plasma membrane.^46^^,^^101^ Using this technology, the same group showed recently that dynamic endoplasmic reticulum-late endosome membrane contact sites can modulate exosome secretion by regulating certain MVB fusion events to the plasma membrane, showing crucial involvement of RAB27.^102^ Moreover, the small GTPase RAL1 was found to control exosome production in 4T1 cells and was further demonstrated to recruit syntaxin 5 (t-SNARE) in *C. elegans*, thereby promoting MVB fusion with the plasma membrane.^47^ Since then, other Ral GTPases, specifically RalA/B, have been implicated, not necessarily in the fusion step, but in MVB homeostasis and exosome secretion by acting directly through PLD1.^48^ Furthermore, to overcome electrostatic repulsion between membranes, the fusion event can be facilitated by bivalent ions.^98^ In fact, MVB fusion with the plasma membrane was demonstrated to depend on calcium in K562 cells.^49^^,^^50^

## Microvesicles

Considerably less is known about the formation of microvesicles (MVs) or ectosomes. Only a few regulators of MV shedding from the plasma membrane have been identified and studied (Table 1). Increasing evidence points to the involvement of small GTPases, such as the Rho family^103^ and ARFs,^51^^,^^52^^,^^53^ by regulating cytoskeletal elements. Activation of ARF6, for example, was found to enhance MV production in melanoma cells, which was further shown to depend on the recruitment or activation of its downstream effectors PLD, ERK, and myosin II light chain kinase (MLCK).^51^ Another study was unable to observe the same effect of ARF6 in HeLa cells, but instead demonstrated that RhoA and its effectors (ROCK, LIMK, cofilin) are involved in MV formation.^52^ Moreover, it is not surprising that, as an upstream regulator of the Rho family, ARF1 was found to govern MV shedding in MDA-MB-231 cells.^53^ Apart from that, acid sphingomyelinase (ASM), as another regulator of ceramide in addition to nSMase, which is involved in exosome biogenesis, has been implicated in MV biogenesis. Initially, it was found that ATP stimulates the production of MVs in microglia,^104^ which relies on the activation of the ionotropic ATP receptor P2X7,^105^ phosphorylation of p38, and ultimately ASM.^54^ Similarly, MV generation in red blood cells was linked to ASM.^55^ Last, the formation of adaptor protein arrestin domain-containing protein 1 (ARRDC1)-mediated MVs was shown to share commonalities with viral gag-induced membrane shedding. Both require the help of TSG101 and VPS4 as well as localization to the plasma membrane, which for ARRDC1 is mediated by its arrestin domain.^56^

## Cargo sorting into EVs

EVs are gaining ground as drug-delivery modalities for a wide range of diseases. They benefit from favorable safety profiles as well as the ability to cross biological barriers and protect their contents from degradation.^8^^,^^9^ While some applications exploit the inherent therapeutic properties of EVs, others manipulate them to deliver a specific cargo.^8^^,^^9^^,^^106^^,^^107^^,^^108^ This manipulation can be realized by endogenous or exogenous means, referring to methods performed pre- or post-EV isolation, respectively.^108^ Exogenous techniques show high efficiencies but may compromise EV integrity and are restricted to smaller payloads (extensively reviewed elsewhere^108^^,^^109^). Endogenous approaches are generally more labor intensive, yet suitable for larger macromolecular cargos.^108^^,^^109^ Endogenous loading techniques mainly encompass genetic engineering of the source cells to overexpress a protein of interest fused to proteins involved in EV biogenesis, consequently exploiting their inherent capabilities to be incorporated into EVs.^106^^,^^108^^,^^110^ In addition, non-genetic source cell manipulation approaches have been successfully established.^110^ In the following section, we will first briefly illustrate the natural loading of macromolecular cargo during EV biogenesis, focusing on protein and nucleic acid cargo. Next, we will discuss current strategies for endogenous bioengineering of source cells to specifically sort therapeutic cargo molecules into or onto EVs by modifying EV biogenesis pathways.

### Protein cargo

EVs carry a broad range of transmembrane proteins, membrane-associated proteins, and luminally loaded soluble proteins. For instance, Hurwitz et al. performed proteomic characterization of EVs derived from 60 different cancer cell lines and identified 6,071 proteins in total, of which 213 were common to all cell types, and only a minority were exclusive to a specific cell source.^111^ Similarly, Kugeratski et al. identified 3,759 proteins in EVs derived from 14 cell lines, of which 642 proteins were unique to different cell types.^112^ Furthermore, Hoshino et al. analyzed 497 EV preparations from cell lines, tissue explants, and plasma, from both mice and humans, and identified homologies in the protein signature of these EVs.^113^ These studies at large reflect the fact that the majority of the EV proteomes are similar and enriched in proteins involved in EV biogenesis. In addition, only a minority of the proteome reflects the cell-type specificity. Importantly, the mechanisms involved in sorting or loading of EV-associated proteins are yet to be determined, but most of these proteins are cell-surface receptors, which could indicate that they originate from plasma membrane budding or shedding.

EVs are highly enriched in various tetraspanins such as CD81, CD63, CD9, CD82, and CD37.^114^^,^^115^ The tetraspanin family comprises proteins that are neither enzyme-linked receptors nor catalytically active receptors, but that may promote the sorting of protein cargos, especially tetraspanin-interacting proteins such as integrins,^116^ ICAM-1,^117^ IGFS-8,^118^ major histocompatibility complex (MHC) class II proteins,^115^^,^^119^ and syndecan.^19^ However, various reports failed to see differences in EV numbers or EV proteomes upon overexpression or silencing of CD63, CD81, or CD9.^120^^,^^121^ Furthermore, based on the cellular localization of tetraspanins, it has been speculated that tetraspanins such as CD63 are exclusively present on EVs of MVB origin, whereas CD81, which is primarily localized on the cell surface, is preferentially sorted into MVs.^12^^,^^57^^,^^122^ This is reflected by the fact that CD63-positive vesicles are CD81 low or negative and vice versa.^12^ In addition to tetraspanins, there are also other scaffolding transmembrane proteins that are associated with EVs, such as flotillin 1 and 2,^123^ IL-6R,^124^ EGFR,^125^ T cell receptor,^126^ chimeric antigen receptor,^127^ Notch receptors,^128^ GPCR receptors,^129^^,^^130^ PD-L1,^131^ TGFB,^132^ and ADAM proteases,^133^ on the surface of EVs. Apart from transmembrane proteins, the surface is also rich in membrane-interacting proteins, especially proteins with glycosylphosphatidylinositol (GPI) anchors, for example, complement-inhibiting proteins DAF and MAC-IP,^134^ as well as cell-surface proteoglycan glypican-1.^135^ In addition, on the inner leaflet, a range of proteins have been identified, including small GTPases, which are involved in the biogenesis and adhere to the inner leaflet by post-translational prenylation.^38^^,^^39^^,^^136^ In addition to prenylated proteins, myristoylated proteins such as BASP-1^137^ and Src signaling kinases^136^ also interact with the inner leaflet and sort into EVs. Similarly, lentiviral gag uses N-terminal myristoylation for loading into viruses or EVs.^138^ Other posttranslational modifications that have been shown to drive cargo sorting are ubiquitination, SUMOlyation,^139^^,^^140^ and phosphorylation.^141^ Other proteins that are abundant in EVs interact with, or are part of, ESCRT complexes such as ALIX, TSG101, and syntenin.^71^ Apart from biogenesis-related proteins, EVs are also enriched with molecular chaperones such as Hsp70, Hsp90, and Hsp20.^142^^,^^143^^,^^144^ Finally, cytosolic proteins, such as actin and tubulin, are also sorted into EVs, most likely upon MV shedding from the plasma membrane.^111^

### RNA cargo

More than a decade ago, multiple studies showed the EV-mediated functional intercellular transfer of RNA.^145^^,^^146^^,^^147^^,^^148^^,^^149^^,^^150^ According to numerous reports using next-generation sequencing and microarray technologies to characterize RNA content in EVs derived from cell culture, tissues, or biological fluids, EVs contain both coding and non-coding RNA species.^151^ Apart from mRNA, EVs are enriched in small RNA species, such as transfer RNAs (tRNAs), microRNAs (miRNAs), small nuclear RNAs (snRNAs), small nucleolar RNAs (snoRNAs), mitochondrial RNAs (mtRNAs), piwi-interacting RNAs (piRNAs), vault RNAs (vtRNAs), and Y RNAs.^96^^,^^151^^,^^152^^,^^153^^,^^154^^,^^155^^,^^156^ Circular RNAs (circRNAs) and fragments of ribosomal RNAs (rRNAs) and long non-coding RNAs (lncRNAs) have also been identified in EVs.^157^^,^^158^^,^^159^^,^^160^^,^^161^^,^^162^^,^^163^

The molecular mechanisms of RNA sorting into EVs are still not fully understood. Current literature indicates that factors like RNA abundance, sequence length, cellular location, or the ability to associate with certain proteins or membrane lipids regulate RNA sorting into EVs.^164^^,^^165^ Generally, sorting mechanisms are classified as active or passive RNA-loading processes.^166^ Passive loading into EVs strongly depends on the intracellular concentration of a certain RNA, and its enrichment in EVs is exclusively source cell conditional.^157^^,^^167^ An active, or selective, loading mechanism of RNA into EVs, on the other hand, is indicated by an enrichment for a certain RNA in EVs that is not necessarily mirrored in the overall RNA content of the source cell.^96^^,^^146^^,^^168^^,^^169^ For instance, preferential enrichment in EVs was reported for 3′ UTR mRNA fragments with regulatory elements and recurring motifs, such as variations of a stem-loop-forming 25-nt “zip code” sequence encompassing a GTGCC core motif and a single miRNA-1289 binding site.^160^^,^^170^ This was also shown specifically for miRNA containing so-called EXOmotifs, with the strongest being CGGGAG, while miRNAs with CELLmotifs, such as AGAAC, CAGU, or AUUA, were retained in the source cells.^171^ Furthermore, 40 miRNA seed sequences were identified as motifs enriched in lncRNA associated with prostate cancer EVs.^172^ Overall, these studies suggest an intriguing mechanism for sorting certain miRNAs into EVs: by using mRNA fragments or lncRNAs as RNA sponges.^170^^,^^172^ Correspondingly, the enrichment of certain sequence motifs in EV RNA could point to a sorting mechanism involving RNA-binding proteins (RBPs) with differential sequence affinities. As there are more than 4,000 RBPs annotated in the human genome at the time of this writing,^173^ and RBPs comprise 25% of the EV protein content,^157^ it can be safely assumed that they play a key role in the active sorting of RNA into EVs. In fact, several RBPs and their selective RNA cargo have been linked to EVs, such as YBX1,^174^^,^^175^^,^^176^^,^^177^ SYNCRIP,^178^^,^^179^ Arc1,^180^ AGO2,^181^^,^^182^ ALIX,^183^ MVP,^184^^,^^185^ hnRNPU,^186^ and ANXA2.^187^^,^^188^ Apart from specific binding of RNA sequence motifs, additional EV sorting signals include RNA or RBP modifications, such as SUMOylation, as reported for miRNA loading into EVs by hnRNPAB1^140^; phosphorylation, as shown for exosomal 5′pppRNA in latent EBV infection^189^; or LC3 conjugation, as described for hnRNPK and SAFB-mediated loading of small ncRNA species during the secretory autophagy pathway.^32^ In addition, RNA association with different membrane lipids during vesicle formation was proposed as another distinct mechanism for selective RNA sorting into EVs.^190^^,^^191^^,^^192^^,^^193^

Even with years’ worth of outstanding research, the genuine complexity of RNA loading mechanisms during EV biogenesis pathways is by far not completely understood. A recent study clearly demonstrated the heterogeneity of RNA content in different EV subpopulations, indicating distinct preferences and, possibly, limitations in loading mechanisms and EV capacity depending on the EV biogenesis pathway.^151^ Therefore, additional efforts to understand EV heterogeneity, especially considering vesicular RNA cargo sorting pathways, are essential. Moreover, recent reports questioned the consensual knowledge of the true biological impact of EV-derived RNA, in particular low-abundant miRNA.^194^ Similarly, awareness was raised in the field to reflect on EV isolation methodologies and the limitations of certain functional assays, especially when working with RNA from EVs produced by transient transfection.^195^^,^^196^^,^^197^ Thus, careful assessment of biological claims and ample use of proper controls is key to unravel the true role and functional impact of EV-derived RNA.

### DNA cargo

While RNA as a cargo nucleic acid in EVs has been extensively studied, there are substantially fewer studies on the biogenesis or clinical significance of EV-associated DNA. To date, DNA species reported associated with EVs include genomic double-stranded DNA (dsDNA),^198^^,^^199^^,^^200^^,^^201^ along with dsDNA-binding histone proteins,^202^ single-stranded DNA (ssDNA),^203^ mitochondrial DNA (mtDNA),^204^^,^^205^ and viral DNA.^206^ Predominantly, EV-associated DNA has been proposed as a putative biomarker in liquid biopsies of cancer patients^198^^,^^207^^,^^208^^,^^209^^,^^210^ or as a tool for non-invasive prenatal diagnostics.^210^^,^^211^ However, EV-associated DNA is mostly sensitive to enzymatic treatment and thus detected mainly on the outside of certain EV subpopulations.^212^ Interestingly, no chromosomal regions have been found to be overrepresented in remaining EV-enclosed DNA, hinting at the absence of a specific loading mechanism for genomic DNA into EVs.^212^ Yet, a nucleosome-associated pattern^212^ of long genomic DNA fragments as well as chromatinized DNA structures^209^ in EVs was observed, suggesting a still elusive mechanism for genomic DNA loading during EV biogenesis.

Conversely, despite evident reports of EV-associated DNA, its presence and biological impact are still highly disputable, as the field often considers EV-associated DNA a contaminant from improper EV purification.^122^ Thus, the authentic role of DNA in EV biogenesis remains to be elucidated.^210^

## Hijacking the EV biogenesis pathway for biotherapeutic cargo loading

EVs are enriched with a variety of biomolecular cargo, including lipids, proteins, and nucleic acids. With the application of new genetic tools, such as RNAi, CRISPR-Cas9, and recombinant DNA technology, in combination with the recent technological advances in EV characterization, the biological phenomena involved in the sorting of cargo molecules into EVs are starting to unravel (Table 1). Thus, by employing current state-of-the-art methods of synthetic biology, we and others have exploited the known biological mechanisms to engineer EVs with a variety of therapeutic cargo.

### Protein cargo loading into EVs

**Figure 2:**
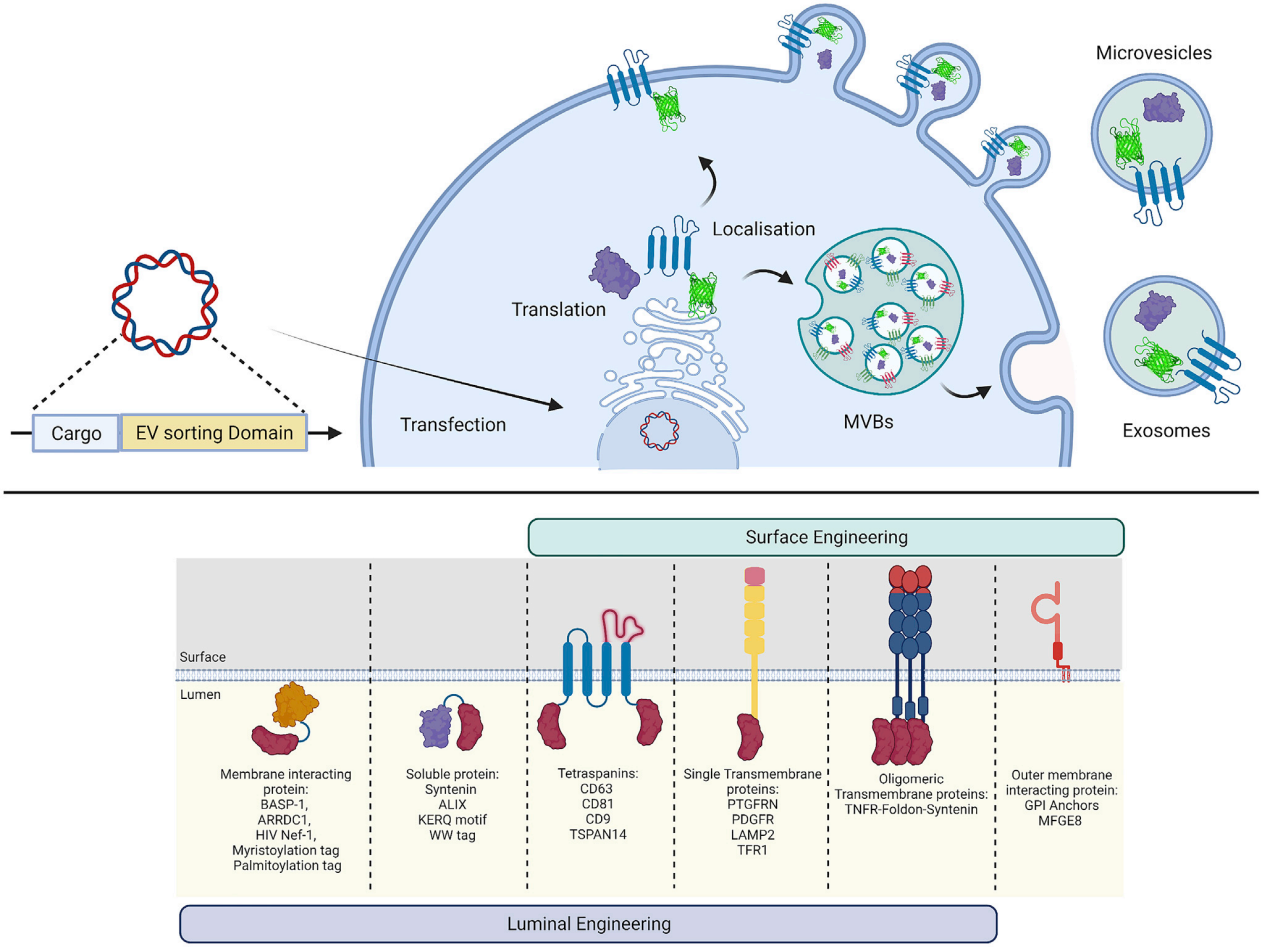
Endogenous EV engineering strategies for therapeutic cargo loading The top illustrates the general principle of producer cell engineering. A genetic expression construct encoding the therapeutic cargo connected to an EV sorting domain is introduced into and expressed by EV producer cells. The therapeutic cargo is sorted into EVs during EV biogenesis. Depending on the nature of the EV sorting domain, engineered EVs originate from the MVB-exosome pathway or microvesicle pathway. The bottom lists means of luminal or surface engineering of EVs, including examples for EV sorting domains successfully used in the field. This figure was created using BioRender.

Protein sorting into EVs is a highly regulated process, and the majority of these proteins are ubiquitously enriched in EVs irrespective of the source cell due to their involvement in EV biogenesis. Therefore, hitchhiking on proteins involved in EV biogenesis serves as an efficient mean of endogenously bioengineering EVs with biotherapeutics. For endogenous loading, the parental cells are genetically engineered to overexpress the desired protein fused to an EV scaffold, which is then incorporated into the secreted vesicles during EV biogenesis (Figure 2).^108^ Myriads of endogenous engineering scaffolds have been tested in the past decade for EV luminal and surface engineering (Figure 2). However, with such a vast diversity of proteins involved in EV biogenesis and cargo loading, identifying versatile strategies to load biotherapeutic cargo into EVs is highly challenging. Importantly, fusion of a certain cargo to one regulator of EV biogenesis may lead to suboptimal loading into EVs, while fusion to another could result in superior engineering performance. Our group has shown this for eGFP, where fusion to ALIX yielded several orders of magnitude lower cargo encapsulation into EVs compared with a fusion to CD63.^121^ Thus, adapting known molecular mechanisms involved in EV engineering is ambitious, owing to their extraordinary complexity, but mindful design and further deepening of our knowledge will result in the development of successful strategies. In the following sections, we discuss current strategies for endogenous EV bioengineering and cargo loading based on native EV biogenesis and touch upon emerging developments and future directions of the EV bioengineering field.

### Luminal-protein loading

EVs are enriched with numerous luminal proteins; however, not all EV proteins can be used for the endogenous engineering of EVs. This is primarily due to loss of functionality upon fusion of the protein of interest to an EV sorting scaffold.^121^ Ideally, the EV engineering scaffold should allow for efficient packaging of multiple copies of proteins without interfering with the EV biogenesis pathway and luminal content of the EVs. Therefore, various studies have systematically compared different EV sorting domains for endogenous engineering using the latest innovations in EV characterization to determine the efficiency at a single-EV level. For instance, we previously systematically compared 12 endogenous EV engineering scaffolds for encapsulating EGFP into EVs and identified the tetraspanins CD63, CD81, and CD9 as the most efficient loading scaffolds.^121^ For instance, CD63 could load 40–60 EGFP molecules per vesicle. Moreover, a recent study by Silva et al. achieved loading of 150 molecules of EGFP per vesicle using TSPAN14 as the EV scaffold.^213^ Interestingly, the subcellular location and resulting EV proteome were largely unaltered upon engineering EVs endogenously with CD63-EGFP fusion proteins.^12^^,^^121^ In addition to tetraspanins, other identified EV sorting domains include syntenin,^121^ ARRDC1,^214^ BASP-1,^137^ syndecan-1,^121^ and HIV-I Nef protein.^215^ Importantly, a direct fusion of the therapeutic protein, to either the N or the C terminus of an EV scaffold, may affect the functionality of the therapeutic protein in the recipient cells.^216^ Therefore, various sophisticated systems have been developed, such as the light-induced dimerization system^216^ and small-molecule-controlled protein association,^217^ which allow for the release of biotherapeutic cargo from the EV scaffold post-encapsulation. Also, the fusion of certain tags to the protein of interest can mediate EV sorting. For instance, insertion of the KFERQ motif can drive loading into Lamp2A-positive EVs.^218^ Similarly, WW-domain-tagged proteins are ubiquitinated upon recognition by Ndfip1 and are efficiently packaged into EVs.^219^ Based on these novel developments in endogenous EV engineering, EVs have been bioengineered for the delivery of CRISPR Cas9,^217^^,^^220^ IκBα superrepressor,^221^ Cre recombinase,^214^^,^^216^^,^^219^ and lysosomal enzymes.^222^ Importantly, all these endogenous EV engineering strategies achieve the labeling of certain EV subpopulations only, as one sorting domain mainly makes use of one of multiple EV biogenesis pathways.^1^ Therefore, exploring different combinations of EV sorting domains to achieve the engineering of a broader EV population will aid in enhancing the therapeutic efficacy of EVs.

### Surface-protein engineering

As mentioned earlier, the EV surface is enriched with various transmembrane proteins or GPI-anchored proteins. These proteins have a variety of effector functions, such as ligands for target cell recognition^223^ and signaling^131^ or receptors for decoying toxins, biologics, and viruses.^133^^,^^224^ For developing advanced therapies using EVs, surface engineering is crucial for achieving targeted delivery, signaling, and decoy function. Similar to the luminal engineering of EVs, various surface engineering scaffolds have been identified through a systematic comparison of EV-associated transmembrane proteins. One of the most widely used scaffolds for the surface display of ligands is Lamp2, a transmembrane protein associated with the endolysosomal pathway.^225^ Importantly, Lamp2b labels vesicle populations originating from the endolysosomal pathway, and engineering efficiency is highly dependent on the producer cell.^121^^,^^226^ Therefore, novel surface engineering scaffolds are much needed to target a broader range of EV subpopulations and ensure high engineering efficiencies independent of their cell sources. In addition to their application in luminal engineering of EVs, tetraspanins can be exploited for the display of protein biologics on the EV surface. The second extracellular loop of tetraspanins is highly modular and facilitates the insertion of ligands for the surface engineering of EVs.^227^ With this strategy we recently dramatically increased EV circulation times *in vivo* by displaying an albumin-binding domain on their surface.^227^

Notably, the use of tetraspanins for surface engineering should be done with caution, as the display of ligands in a closed-conformation extracellular loop may affect the functionality. Furthermore, the display of large ligands in the extracellular loop can have a negative impact on the sorting of the fusion protein into EVs.^121^ To overcome this limitation, a fifth transmembrane domain has been added to either the N or the C terminus of tetraspanins, or the fourth transmembrane domain is deleted to facilitate the display of larger ligands on the EV surface.^226^^,^^228^ Apart from the tetraspanin protein family, PTGFRN was identified as a scaffold for surface engineering of EVs and achieved loading of up to 1,000 engineered molecules per EV.^137^ Importantly, PTGFRN interacts with CD9 and CD81, and the use of this scaffold may target CD9 and CD81 EV subpopulations.^229^ Similarly, PDGF, another EV-associated transmembrane protein, has been used for EV engineering.^230^ Apart from transmembrane proteins, GPI anchors^231^ and phosphatidylserine-binding proteins such as MFGE8^232^ can also facilitate surface engineering of EVs. Notably, the association of these scaffolds with an EV surface is reversible and dependent on phospholipid composition, which may limit the engineering efficacy. EV-associated luminal proteins have also been used for surface engineering of EVs. We recently described one such approach in which transmembrane proteins, upon fusion with syntenin and an oligomerization domain, enabled efficient EV surface engineering, while the EV surface proteome was largely unaltered.^226^ Importantly, this strategy proved to be more efficient for the surface display of cytokine receptors than for the use of other scaffolds, such as PTGFRN, MFGE8, PDGF, or CD63.^226^

### Nucleic acid cargo loading

**Figure 3:**
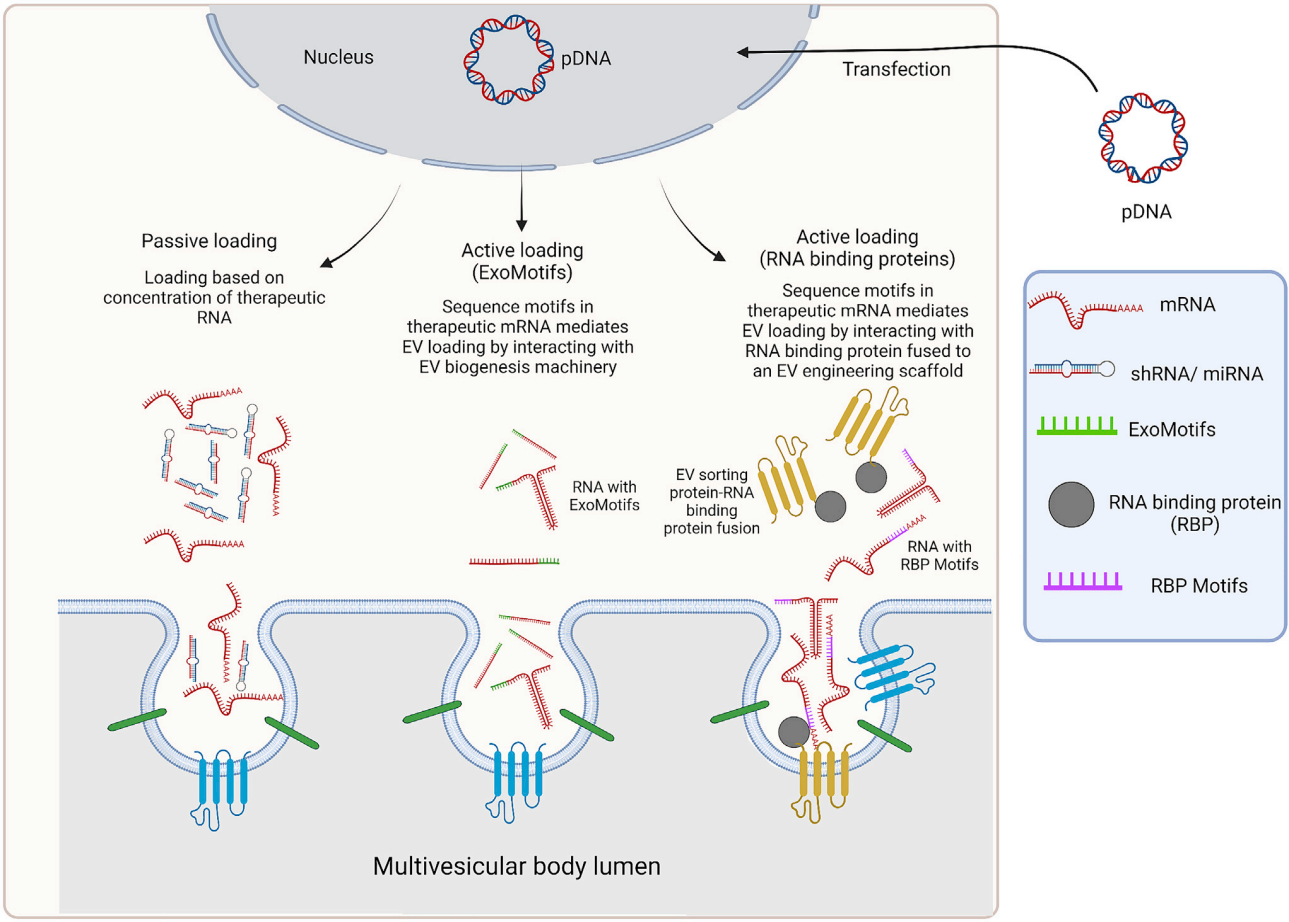
Strategies for nucleic acid loading into EVs RNA species are sorted into EVs either passively, triggered by high local abundance and membrane proximity, or specifically, by means of active sorting. Active sorting processes are initiated by the presence of defined motifs in the RNA sequence, such as EXOmotifs or RBP motifs, that either associate with luminal membranes during MVB formation or are recognized by RNA-binding proteins (RBPs), which in turn are incorporated into EVs during biogenesis. These natural sorting pathways during EV biogenesis can be exploited for bioengineering strategies to achieve targeted loading of therapeutic RNA cargos into EVs. This figure was created using BioRender.

After the discovery of functional RNA transfer via EVs,^145^ tremendous efforts were invested to create bioengineered EVs as nanosized biomimetic delivery agents for therapeutic nucleic acid cargo. While small RNA species can nowadays be efficiently and successfully encapsulated into post-purified EVs by exogenous loading approaches,^225^^,^^233^^,^^234^ the development of efficient and robust endogenous loading required for larger nucleic acid cargos has proven significantly more difficult. As exact sorting mechanisms of any nucleic acid species into EVs remained unclear, the first approaches to endogenous EV bioengineering for nucleic acid loading were based on overexpression of sequence-optimized miRNAs, small interfering RNAs (siRNAs), and other small RNAs,^190^^,^^235^^,^^236^^,^^237^ as well as therapeutic mRNAs (Figure 3).^238^^,^^239^ However, as knowledge about specific RNA sorting mechanisms deepened, the resulting exploitation of EV biogenesis pathways has greatly accelerated the development of EV bioengineering strategies. For instance, siRNA sequences incorporated into a Dicer-independent pre-miRNA stem loop (pre-miR-451) showed enhanced loading efficiency using the miRNA sorting machinery into EVs.^240^ Also, this approach of siRNA delivery dramatically decreased the siRNA dose needed in target cells for effective gene silencing.^240^ For EV loading of nucleic acid cargo, the endogenous loading strategies for therapeutic proteins as discussed previously have been adapted by fusing a nucleic acid-binding protein to an EV-sorting protein. Transient or stable co-expression of the fusion protein with the nucleic acid displaying the compatible binding motif leads to nucleic acid binding and efficient loading into the EVs during biogenesis (Figure 3). This methodology has been successfully employed for small RNAs,^230^^,^^241^^,^^242^ as well as longer RNA species, such as mRNA.^214^^,^^243^^,^^244^ Target therapeutic mRNA expression in EV source cells also leads to therapeutic protein expression and passive protein loading into EVs.^245^ Thus, special care needs to be applied when evaluating cargo mRNA functionality upon delivery of engineered EVs to recipient cells.^245^ Consequently, in an experimental setting, the co-delivery of passively loaded protein needs to be properly controlled for, while in a therapeutic setting, co-delivery of mRNA and therapeutic protein can be favorable. A recent approach addressing this methodological issue elegantly combined endogenous and exogenous EV loading strategies.^246^ Here, they expressed a DNA aptamer sealing target mRNA translation in producer cells while being efficiently loaded into EVs by a CD9-zinc finger fusion protein.^246^ To disable DNA aptamer binding, purified mRNA/DNA aptamer-loaded EVs were electroporated to encapsulate a Klenow fragment exonuclease, degrade the aptamer, and enable mRNA translation in recipient cells *in vitro* and *in vivo*.^246^

As increasing numbers of strategies for EV endogenous engineering platforms for therapeutic RNA delivery emerge, the scientific discussion about capacity limitations of EVs as a drug-delivery tool becomes more pressing. For instance, naturally sorted rRNA and snRNA have been identified at more than one copy per EV, while full-length mRNA copies have been reported at a maximum figure of 1 in 1,000 EVs.^151^^,^^165^ Moreover, full-length mRNA transcripts longer than 1,000 nt are almost absent in small EVs and detectable only in larger vesicles.^151^ Thus, the question arises as to which of the biologically very heterogeneous EV populations have the physical capacity and means to be endogenously loaded with full-length, functional longer RNA species? Furthermore, once acquired, how can the field translate this knowledge into technology development? Currently, there are neither binding official agreements on nucleic acid quantification or reporting methods nor a gold standard in EV purification methodology.^18^^,^^164^^,^^247^ EV production and purification are subject to informed methodological decisions of the respective researcher, and thus a fair comparison of RNA loading figures from the literature seems nearly impossible. However, efficiencies of up to one selectively loaded mRNA copy per EV have been reported when passive loading of protein, attached to the mRNA or translated from it, was suppressed.^246^ This observation points to a promising avenue toward improving RNA loading strategies. In addition, and highly remarkably, as biomimetic vehicles for RNA delivery, EVs performed much more efficiently than state-of-the-art lipid formulations.^237^^,^^240^^,^^248^ Thus, further unlocking mechanisms of EV biogenesis for the development of safe, elegant, and efficient engineering strategies for nucleic acid delivery via EVs will tremendously accelerate the nanomedicine and gene therapy fields for a plethora of applications.

## Non-genetic source cell modifications for endogenous EV loading

Genetic engineering for endogenous cargo loading into bioengineered EVs bears certain safety risks for their clinical applicability.^110^^,^^249^ Thus, non-genetic means of source cell modifications exploiting EV biogenesis pathways are valuable to the field. Current methodologies include cellular metabolic labeling and intrinsic cell membrane modifications.^110^^,^^249^ For instance, glycans of EV producer cells were metabolically engineered to incorporate active azides into the membranes of secreted EVs, which were then combined with bioorthogonal click conjugation to modulate the EV characteristics after EV purification.^250^^,^^251^ Furthermore, the treatment of endothelial cells with ethanol led to increased sorting of pro-angiogenic miRNAs and lncRNAs into secreted EVs.^252^^,^^253^ Another non-genetic loading technique is the recycling of externally provided EV cargos. Interestingly, phagocytic cells have been reported to incorporate superparamagnetic iron oxide nanoparticles along with therapeutic agents into their EVs by phagocytosis when supplied with the EV cargo through simple addition to the growth medium.^254^^,^^255^ Alternatively, fusing the EV source cell membrane with synthetic membrane fusogenic liposomes containing hydrophobic compounds was described as another method to load cargo into secreted EVs.^256^^,^^257^ As large-scale EV production is still a significant bottleneck in clinical translation of EV therapeutics, non-genetic approaches to produce sufficient yields of therapeutically active EVs can be indispensable additions to existing EV bioengineering strategies.^239^^,^^258^^,^^259^^,^^260^ Methodologies to boost EV production and secretion include, but are not limited to, metabolic changes in the source cells by defined medium compositions,^258^^,^^259^^,^^260^ 3D cultures in bioreactors,^253^^,^^258^^,^^261^^,^^262^ and physical stimulation.^239^^,^^263^^,^^264^

## Conclusions

The field of EVs has seen an immense transformation in the past few decades, from being their regarded as garbage bags to now being regarded as essential mediators in intercellular communication. Owing to their unique ability to transfer macromolecules across cells and biological barriers, EVs are considered a rising star in the field of drug delivery. Notably, EVs outcompete the majority of the synthetic delivery vectors in terms of better efficacy, extrahepatic delivery, and much lower toxicity. Overall, these developments have led to the initiation of various clinical trials using EVs as a therapeutic intervention. Intriguingly, several trials report therapeutic activity and, most importantly, no toxicity has been observed overall in the study participants. These reports on ongoing phase 1 clinical trials are highly promising, and it is hoped they will lead to a progression to phase 2/3 placebo-controlled settings soon. However, although technologies for large-scale and Good Manufacturing Practice (GMP)-grade culturing of cells exist, clinical manufacturing of EVs is still an unaddressed territory. This is primarily hindered by a lack of technologies that allow for large-scale production of highly pure EVs without affecting their integrity and biophysical properties. A particular challenge for the purification of EVs from the extracellular environment is its enrichment in apoptotic bodies, protein aggregates, and ribonucleic complexes, which display features similar to those of EVs, such as size or density, and thus potentially co-purify with EVs using current technologies. Another obstacle, which is largely unaddressed, is the GMP/Good Clinical Practice (GCP)-grade storage while conserving the therapeutic effect of EVs. Acquisition of more in-depth knowledge about EV stability and development of purification technologies for enriching specific EV populations will be key to achieve a smooth translation from bench to bedside in the future.

## Funding

This project has received funding from the European Research Council (ERC) under the consolidator grant (proposal n° 101001374) and the European Union's Horizon 2020 research and innovation programme (grant agreement n° 825828). The project was further supported by the Swedish Research Council (agreement n° 4-258/2021), Cancerfonden (agreement n° 4-511/2022), and The Strategical Research Foundation (SSF) Industrial Research Centre (agreement n° IRC15-0065).
